# Gene Therapy Overexpressing Neuregulin 1 Type I in Combination With Neuregulin 1 Type III Promotes Functional Improvement in the SOD1^G93A^ ALS Mice

**DOI:** 10.3389/fneur.2021.693309

**Published:** 2021-09-22

**Authors:** Guillem Mòdol-Caballero, Mireia Herrando-Grabulosa, Sergi Verdés, Belén García-Lareu, Neus Hernández, Isaac Francos-Quijorna, Rubén López-Vales, Assumpció Bosch, Xavier Navarro

**Affiliations:** ^1^Department of Cell Biology, Physiology and Immunology, Institute of Neurosciences, Universitat Autonoma de Barcelona, Barcelona, Spain; ^2^Centro de Investigación Biomédica en Red Sobre Enfermedades Neurodegenerativas (CIBERNED), Barcelona, Spain; ^3^Department of Biochemistry and Molecular Biology, Institute of Neurosciences, Universitat Autònoma De Barcelona, Barcelona, Spain; ^4^Unitat Mixta UAB-VHIR, Vall d'Hebron Institut de Recerca (VHIR), Barcelona, Spain

**Keywords:** amyotrophic lateral sclerosis, neuregulin 1, ErbB receptors, motoneuron, neuromuscular junction, spinal cord

## Abstract

Amyotrophic lateral sclerosis (ALS) is a fatal neurodegenerative disease affecting the neuromuscular system for which currently there is no effective therapy. Motoneuron (MN) degeneration involves several complex mechanisms, including surrounding glial cells and skeletal muscle contributions. Neuregulin 1 (NRG1) is a trophic factor present particularly in MNs and neuromuscular junctions. Our previous studies revealed that gene therapy overexpressing the isoform I (NRG1-I) in skeletal muscles as well as overexpressing the isoform III (NRG1-III) directly in the central nervous system are both effective in preserving MNs in the spinal cord of ALS mice, opening novel therapeutic approaches. In this study, we combined administration of both viral vectors overexpressing NRG1-I in skeletal muscles and NRG1-III in spinal cord of the SOD1^G93A^ mice in order to obtain a synergistic effect. The results showed that the combinatorial gene therapy increased preservation of MNs and of innervated neuromuscular junctions and reduced glial reactivity in the spinal cord of the treated SOD1^G93A^ mice. Moreover, NRG1 isoforms overexpression improved motor function of hindlimb muscles and delayed the onset of clinical disease. However, this combinatory gene therapy did not produce a synergic effect compared with single therapies, suggesting an overlap between NRG1-I and NRG1-III activated pathways and their beneficial effects.

## Introduction

Amyotrophic lateral sclerosis (ALS) is a fatal neurodegenerative disorder characterized by the loss of upper and lower motoneurons (MNs) leading to progressive paresis, muscle atrophy and death, often due to respiratory failure (1–3). In most patients the cause of ALS is unknown or sporadic (sALS), although some inherit familiar forms of the disease (fALS), associated with several alterations in genes (4), such as superoxide dismutase 1 (SOD1) (5), hexanucleotide repeat expansions in chromosome 9 open reading frame 72 (C9orf72) (6, 7), TAR-DNA binding protein (TDP-43) (8, 9), and DNAJC7 (10). During the last decades, progress has been made on identifying the pathophysiological mechanisms underlying the development of ALS (2). It is likely that the disease has a multifactorial cause, contributed by complex interactions between genetic and molecular pathways (11). Among these, mutant SOD1 has been described to form aggregates leading to an impaired protein degradation and autophagy, exerting toxic effects, and causing MN degeneration (12). Furthermore, evidence has also shown that neighboring cells, such as microglia, astrocytes and interneurons participate in the SOD1-related ALS pathogenesis (13–16). Previous studies have shown that disruption of neuromuscular junctions (NMJs) and axonal degeneration precedes MN death (17, 18), whereas a toxic role of skeletal muscle fibers was also reported (19).

Among the animal models carrying different ALS-related mutations, the most extensively used is the transgenic mouse with the human mutation of a glycine to alanine conversion at the 93rd amino acid of the SOD1 gene (SOD1^G93A^) (20, 21). These mice show various important clinical and pathological characteristics of both the familial and the sporadic forms of the human ALS (18, 21, 22). In addition, further relevance of this model comes from the finding that accumulation of wild-type SOD1 protein may also cause MN disease in mice (23).

Regrettably there are no effective treatments for ALS, and the current standard of care is limited to symptomatic and palliative therapies (24). Only two drugs have been approved for ALS, riluzole, that extends patients survival for a few months, and edaravone, proposed to function by reducing oxidative injury in central neurons.

Interestingly, the ALS field has entered a thrilling era of therapeutic development as shown by the potential of gene therapies in the recent years (3, 25, 26). This approach allows to specifically deliver a gene of interest overcoming the limitation of large proteins crossing the blood-brain barrier (27). Particularly, adeno-associated vectors (AAV) have the ability to provide sustained gene expression without risk for insertional mutagenesis. Moreover, viral transgene expression may target specific cell types by using viral vectors with particular tropism and employing promoters that induce selective and defined gene expression (28).

Notably, spliced forms of Neuregulin 1 (NRG1) yield neurotrophic factors that play essential roles in promoting MN survival, supporting axonal, and neuromuscular development and maintenance (29–35). NRG1 is expressed by spinal MNs, with particular localization at the subsurface cisterns close to postsynaptic sites of cholinergic C synapses (36, 37). Strikingly, alterations in the NRG1 and ErbB receptors have been linked to ALS since loss-of-function mutations of ErbB4 is the cause of a form of autosomal-dominant ALS (38). In this sense, previous studies indicated alterations of the expression of NRG1 isoforms in both ALS patients and SOD1^G93A^ mice, with downregulation of NRG1 type III and upregulation of NRG1 type I in the spinal cord (39, 40). In a recent study, we showed that treatment with an AAV coding for the full-length form of NRG1-III improves motor function of hindlimb muscles and increases MN survival in SOD1^G93A^ mice (40). Importantly, NRG1-I expressed by the Schwann cells has a crucial role in axonal regeneration and remyelination (41), in development of NMJs (31) and in muscle reinnervation (42). Thus, overexpression of NRG1-I causes neuromuscular functional improvement, maintaining muscle innervation, and also increasing MN survival in SOD1^G93A^ mice (43). Therefore, considering these recent findings, in the present study our aim was to combine these two gene therapies overexpressing NRG1-III in the spinal cord to preserve MNs and NRG1-I in muscles to maintain their innervation, searching for a synergic effect.

## Materials and Methods

### Animals and Study Design

Transgenic B6SJL-Tg(SOD1^*^G93A)1Gur mice expressing the mutation G93A in the human SOD1 gene were obtained from the Jackson Laboratory (Bar Harbor, ME). Non-transgenic wild-type (WT) littermates were used as controls. Mice were maintained by crossing transgenic males with F1 hybrid females originally obtained from Janvier Laboratories (France). Transgenic mice were identified by analyzing tissue samples from the tail. Animals were maintained under standard conditions and handled in accordance with the guidelines of the European Union Council (Directive 2010/63/EU) and Spanish regulations on the use of laboratory animals. The Ethics Committee of the Universitat Autònoma de Barcelona approved the corresponding experimental procedures.

In this study we included three groups of B6xSJL female mice, one of WT mice and two of SOD1^G93A^ mice, administered at 7 weeks of age with AAV coding either for NRG1-III and NRG1-I or the respective mock vectors. The following mice groups were used for functional analysis: WT mice (*n* = 6 females), SOD Mock mice (*n* = 18 females), SOD NRG1-I + NRG1-III FL mice (*n* = 11 females). Then, mice were distributed in two subgroups at 16 weeks, one for analysis of mRNA expression: WT (*n* = 3 females), SOD Mock (*n* = 4 females), SOD NRG1-I + NRG1-III (*n* = 5 females), and another for histological studies: WT (*n* = 3 females), SOD Mock (*n* = 5 females), SOD NRG1-I + NRG1-III (*n* = 6 females).

A time-chart showing the AAV administration and the follow-up made during progression of the disease in the SOD1^G93A^ mice can be found in the Supplementary Figure 1.

### Virus Production and Administration

The AAV vectors used have been previously described (40, 43). The full length sequence of the NRG1-III or the extracellular domain of the NRG1-I isoforms containing a HA-tag were cloned between AAV2 ITRs under the regulation of the ubiquitous CMV promoter or the human muscle desmin promoter, respectively. To stabilize mRNA expression, the woodchuck hepatitis virus responsive element (WPRE) was added at 3′ of the constructs. AAV8 and AAVrh10 vectors were generated using the triple transfection system in HEK293-AAV cells of expression and capsid plasmids (Rep2Cap8 or Rep2Caprh10, provided by J.M. Wilson, University of Pennsylvania, Philadelphia, USA) and adenoviral helper sequences contained in pXX6 (44). Viral particles were purified in an iodixanol gradient, and titred using the Picogreen (Invitrogen) system (45) by the Viral Production Unit of UAB-VHIR (http://www.viralvector.eu). AAV8 vector coding mock or NRG1-I, and AAVrh10 virus coding mock or NRG1 type III were produced as in previous studies (40, 43).

For the combined therapy, intravenous administration of 1x10^12^ vg of AAV8-desmin-NRG1-I and intrathecal administration of AAVrh10-CMV-NRG1-III were performed in 7 weeks old mice. For intrathecal injection, under anesthesia with ketamine/xylazine (100/10 mg/kg i.p.), the spine was surgically exposed and 10 μl of viral vector (1 × 10^11^ vg of AAVrh10-CMV-Nrg1-III vector or mock vector) were delivered between lumbar vertebrae L3 and L4 into the cerebrospinal fluid (CSF) with a 30G needle attached to a Hamilton syringe, as previously described (46). Suitable injection into the intrathecal space was confirmed by the animal's tail flick.

### Electrophysiological Tests

Motor nerve conduction tests were performed as previously described (18, 43) by stimulating the sciatic nerve and recording the compound muscle action potential (CMAP) from tibialis anterior (TA), gastrocnemius (GM), and plantar interossei (PL) muscles at 9, 12, 14, and 16 weeks of age. Motor unit number estimation (MUNE) was calculated following previously described protocol using an incremental technique (18, 43). Motor evoked potentials (MEP) recorded from TA and GM muscles were studies to determine the central descending pathways as previously reported (18, 43).

### Locomotion Tests

To evaluate motor coordination and strength of the animals the rotarod test was conducted by placing mice onto the rod turning at a speed of 14 rpm, weekly from 9 to 16 weeks of age (43).

### Nucleic Acids Extraction and Real Time PCR

Mice were sacrificed by decapitation after deep anesthesia at 16 weeks of age. L4-L5 spinal cord segments and gastrocnemius muscles were harvested and frozen. Total RNA was extracted with QIAzol Lysis Reagent (QIAGEN) and 1 μg per sample was reverse transcribed using the iScript™ Reverse transcriptase and Reverse Transcription Supermix (Bio-Rad). The reverse transcription cycle conditions were 25°C for 5 min, 42°C for 20 min and 95°C for 1 min. We analyzed mRNA expression of Nrg1-I and Nrg1-III, by means of specific primer sets: Nrg1-I Fwd TGGGAACGAGCTGAACCGCA; Nrg1-I Rev TCCAGAGTCAGCCAGGGATG; Nrg1-III Fwd TTCCCTTCTCCAGCTCGGACC; Nrg1-III Rev GTCCCAGTCGTGGATGTAGATG. Mouse 36B4 was used to normalize the expression levels (m36B4 Fwd ATGGGTACAAGCGCGTCCTG; m36B4 Rev AGCCGCAAATGCAGATGGAT. Messenger RNA analyses were performed by real-time PCR (Bio-Rad CFX384 real-time PCR system). The thermal cycling conditions started with 5 min polymerase activation at 95°C, followed by 45 cycles of 15s at 95°C, 30s at 60°C, 30s at 72°C and 5s at 65°C to 95°C (increasing 0.5°C every 5s). Melting curves were analyzed by monitoring the SYBR Green decrease after extension. The Pfaffl method was used for quantification relative to controls using the 36B4 gene for mRNA or Cyclophilin for DNA (47).

Spinal cord and GM muscle DNA was extracted with proteinase K (0.1 mg/ml; Sigma), followed by phenol/chloroform extraction. Real time primers for the housekeeping gene cyclophilin B, Desmin-NRG1-I or CAG-Nrg1-III constructs, with the forward primer anneals with the promoter sequence and the reverse primer with each Nrg1 isoform, are the following: mCycloB Fwd TCAACCTCTCCTCTCCTGCC; mCyclo Rev: GGTTTCTCCACTTCGATCTTGC; Desmin GGGTTTGGGGTTCTGAATGTG; Nrg1-I Rev CGGTCCTTCTTCTTGCCCTT; CMV AGAACCCACTGCTTACTGGC; Nrg1-III CGGTCCTTCTTCTTGCCCTT. Viral genome copies/cell were calculated using a standard curve generated from known amounts of a plasmid DNA containing a CMV-Nrg1-III or Des-Nrg1-I sequences or a 500 bp cyclophilin PCR product (CyclophilinB-Fwd5617: CATGCCTATGGTCCTAGCTT and CyclophilinB-Rv6141) purified by Geneclean (Q-Biogene) in 10 ng per ml of salmon's sperm DNA (Sigma) and assuming that 1 mg of mouse genomic DNA contains 3x10^5^ haploid genomes (46).

### Histological Analysis

Other subgroups of mice were perfused with 4% paraformaldehyde in PBS also at 16 weeks of age. Lumbar spinal cord and the GM muscle were obtained. Spinal cords were post-fixed and cryopreserved in 30% sucrose in PBS. GM muscles were directly cryopreserved in 30% sucrose in PBS.

For spinal MN counting, transverse sections were cut at 20 μm thickness with a cryotome (Leica, Germany) and serially collected. Slides containing L4-L5 spinal cord sections separated 100 μm were stained with cresyl violet. Motoneurons were identified in the lateral side of the ventral horn and counted if fulfilling the criteria of having diameter larger than 20 μm, polygonal shape and prominent nucleolus.

For astrocyte and microglia labeling, other sections of L4-L5 segments were immune-labeled with primary antibodies anti-ionized calcium binding adapter molecule 1 (Iba-1, 1:1,000; 019-19741, Wako, Japan), and anti-glial fibrillary acidic protein (GFAP, 1:1,000; 130300, Invitrogen, USA), respectively (40, 43). Images were visualized under a fluorescence microscope (Nikon Eclipse NI, Japan). The integrated density of Iba1 and GFAP was measured by centering the ventral horn using ImageJ software.

For NMJ labeling, longitudinal sections of the GM muscle were cut at 60 μm thickness and collected in sequential series in Olmos solution. Sections were processed for immunolabeling with primary antibodies anti-synaptophysin (1:500; AB130436, Abcam, UK) and anti-neurofilament 200 (NF200, 1:1,000; AB5539, Millipore, USA) as previously reported (43). Then, incubated with Alexa 594-conjugated secondary antibody (1:200; A11042, Invitrogen, USA) and Alexa 488 conjugated alfa-bungarotoxin (1:200; B13422, Life Technologies, USA). Images were visualized and captured under scanning confocal microscopy (LSM 700 Axio Observer, Carl Zeiss, Germany). NMJs were classified depending on whether the endplate was fully occupied (innervated), partially occupied or vacant (denervated). At least 100 endplates were analyzed per muscle.

### Immunoblot Analysis

Snap-frozen lumbar spinal cord samples were sonicated and homogenized in RIPA lysis buffer plus a mixture of protease inhibitors (Millipore). Total protein concentration was determined by BCA Protein Assay Kit (ThermoScientific). Protein separation was performed on 10% SDS-polyacrylamide electrophoresis gel (VWR Life Science), transferred to PVDF membranes (GE Healthcare) and immunoblotted. The following primary antibodies were used: rabbit anti-phospho Erk1/2 (1:500, #9101Thr202/Tyr204, Cell Signaling), total Erk (1:500; #9102, Cell Signaling), rabbit anti-phospho Akt (1:500, Ser473 #9271, Cell Signaling), total Akt (1:500; #9272 Cell Signaling), and rabbit anti-GAPDH (1:1,000; #14C10, Cell Signaling). The secondary antibodies were anti-rabbit HRP-conjugated secondary antibody (1:10,000; Dako) and Westar Eta C Ultra 2.0 ECL substrate (CYANAGEN). Image density was analyzed with Image Lab^TM^ software (Bio-Rad). At least 3 animals per group and treatment and 3 different blots were quantified for each protein analyzed.

### Statistical Analysis

To avoid bias, experiments were performed by researchers blinded regarding the treatment received by each group, and mice were randomly distributed in groups. Sample size was selected according to previous observations in our lab. Data are shown as mean ± SEM. Results of motor nerve conduction and locomotion tests were analyzed with repeated measurements ANOVA, followed by Bonferroni *post-hoc* test when necessary. Results of MUNEs and MEPs were analyzed with t-Student test. For clinical disease onset the Log-rank (Mantel-Cox) test was applied. Results of histological and molecular biology analyses were compared using t-Student or ANOVA with Tukey's *post-hoc* test. A difference was considered significant if *p* ≤ 0.05.

## Results

### NRG1-I and NRG1-III Overexpression in Skeletal Muscles and Spinal Cord

**Figure 1:**
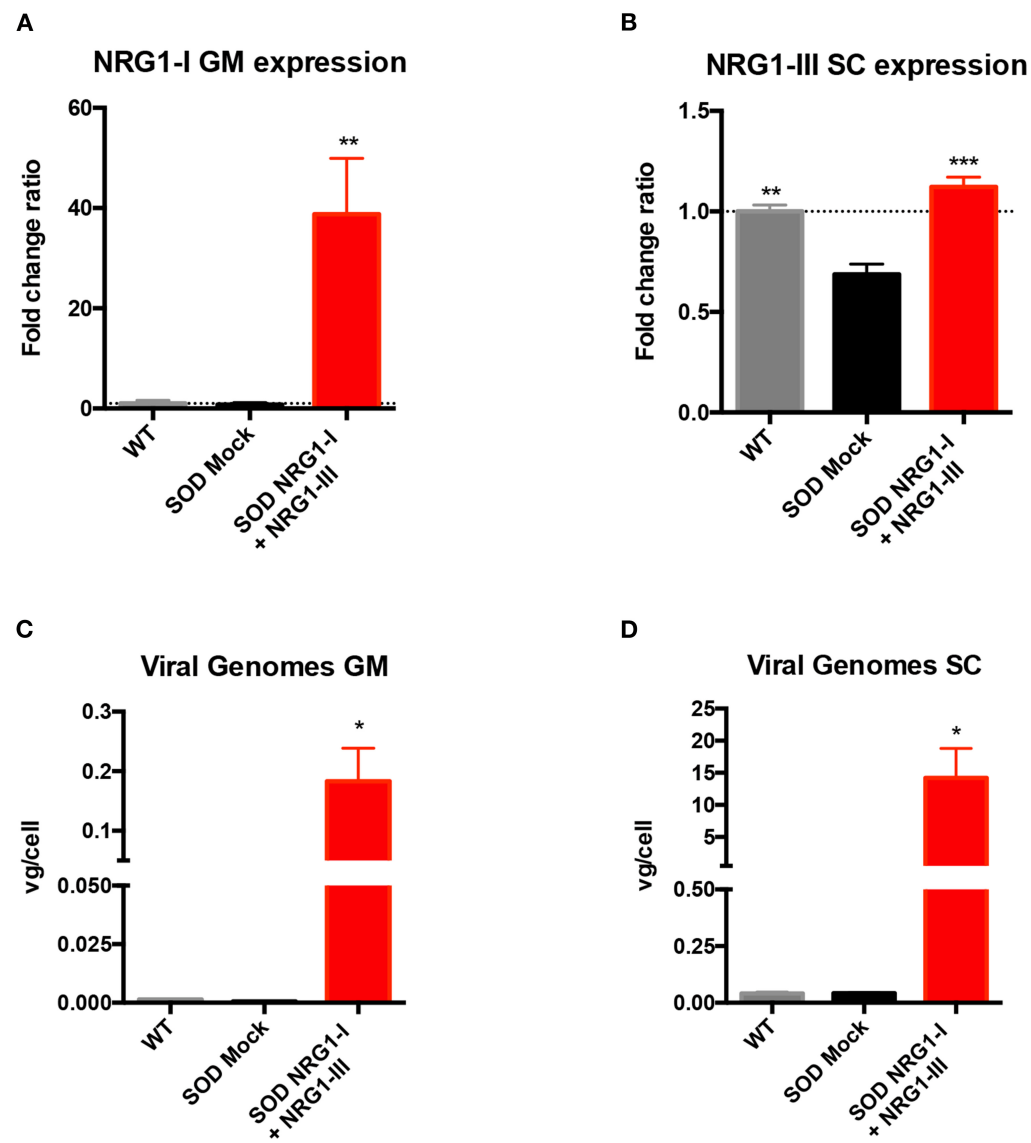
Combined-gene therapy upregulates NRG1-I in the GM muscle and NRG1-III in the spinal cord. **(A)** mRNA expression analysis indicated an overexpression of NRG1-I in the GM muscle upon treatment in the SOD1^G93A^ mice. **(B)** In the lumbar spinal cord, NRG1-III overexpression rescues NRG1-III mRNA levels similar to WT group, whereas it is downregulated in the SOD1^G93A^ mock group. **(C,D)** Viral genome analysis reveals that intravenous and intrathecal AAV administration were effective in targeting both GM muscle **(C)** and Spinal Cord **(D)**. [(*n* = 3 WT, 3-4 SOD Mock, 4-5 SOD NRG1-I + NRG1-III mice per group, one-way ANOVA, ***p < 0.001, **p < 0.01, *p < 0.05 vs. SOD Mock mice]. Data are shown as mean ± SEM.

Taking into account the positive results obtained from the application of either NRG1-I or NRG1-III gene therapies (40, 43), we aimed to test whether a combined therapy overexpressing both isoforms at different sites of the motor unit could produce a synergic effect on the SOD1^G93A^ mice. Vector injections were performed at the same intervention, previous to disease onset (at 7 weeks of age). mRNA analysis at the advanced-stage of the disease (16 weeks) showed that NRG1-I expression was increased in the GM muscle (38.77 ± 11.18) of treated in comparison with the control SOD1^G93A^ mice (0.76 ± 0.41) (Figure 1A), and NRG1-III was increased in the spinal cord (1.12 ± 0.04) compared to the respective SOD1^G93A^ control mice (0.68 ± 0.05) (Figure 1B). In agreement with these results, we detected 0.18 ± 0.05 viral genomes (vg)/cell of Desmin-NRG1-I in the GM and 14.21 ± 4.58 vg/cell of CMV-NRG1-III in the spinal cord of the treated SOD1^G93A^ mice (Figures 1C,D). As expected, qPCR did not detect changes in NRG1-I mRNA in the spinal cord of treated SOD1^G93A^ animals, since AAV8 does not cross the BBB and the desmin promoter is not expressed in spinal cord. Low NRG1-III expression was detected in GM muscles of treated SOD1^G93A^ that can be explained by CSF drainage to the periphery, although five times lower than by intravenous administration of the muscle specific promoter vector (Supplementary Figure 2).

### Combined NRG1 Gene Therapy Preserves Motor Function in SOD1^G93A^ Mice

**Figure 2:**
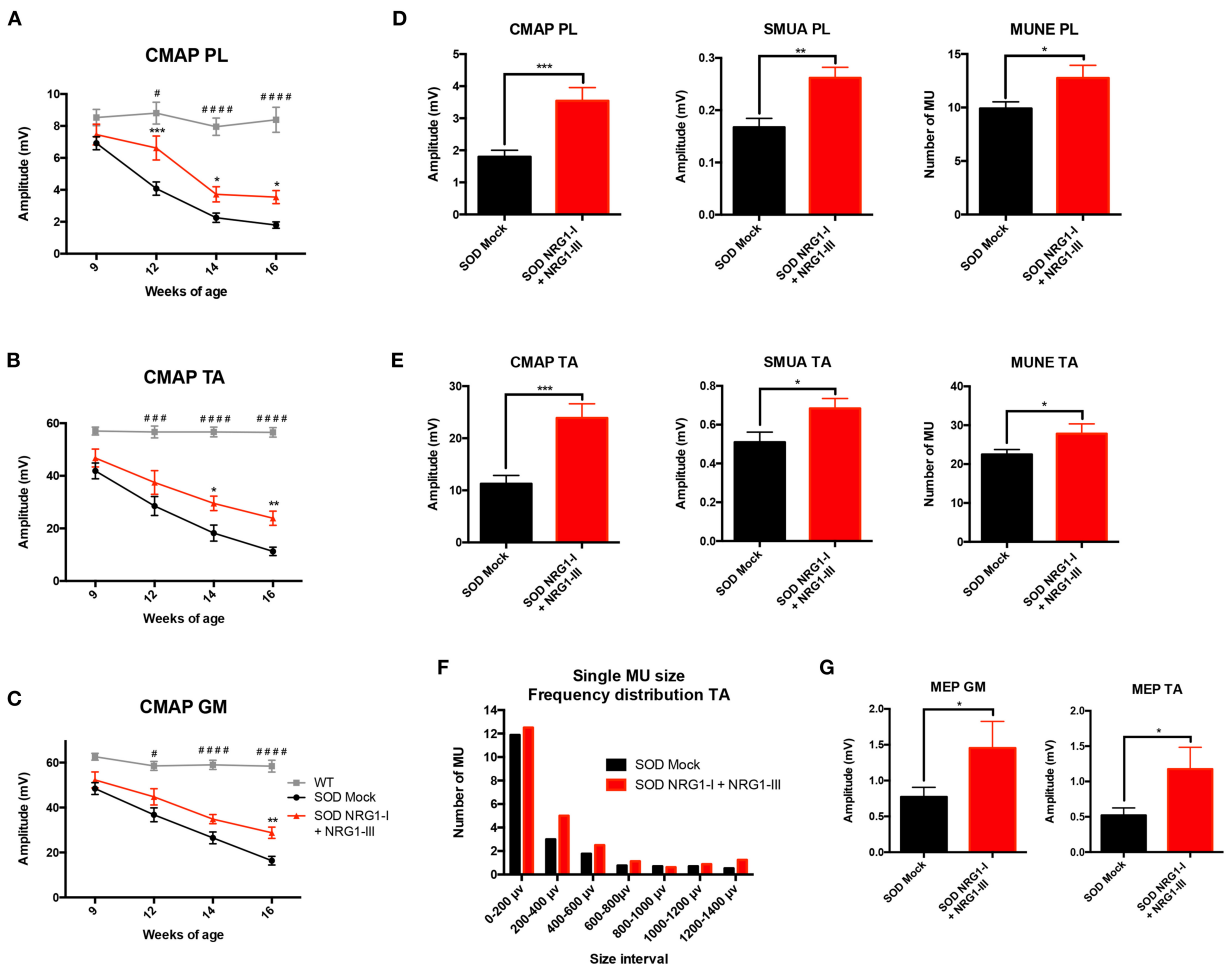
Combined-gene therapy produces functional improvement of the SOD1^G93A^ mice. Electrophysiological tests demonstrate that AAV-NRG1-I and AAV-NRG1-III FL injections promoted a significant preservation of the CMAP amplitude of plantar **(A)**, tibialis anterior **(B)**, and gastrocnemius **(C)** muscles in the SOD1^G93A^ mice (*n* = 6 WT, 16-18 SOD Mock, 7-9 SOD NRG1-I + NRG1-III mice per group, *p < 0.05 vs. SOD Mock mice; ^####^*p* < 0.0001, ^###^*p* < 0.001, ^#^*p* < 0.05 vs. WT mice). Electrophysiological estimation of motor unit number (MUNE) and mean amplitude of single motor unit potential (SMUA) of plantar **(D)** and tibialis anterior **(E)** muscles show a general preservation of motor units induced by the combined therapy, confirmed by the frequency distribution **(F)**. **(G)** The combined therapy increased the amplitude of the MEPs, indicating enhanced connection between upper and lower MNs. (*n* = 14-21 SOD Mock, 5-9 SOD NRG1-I + NRG1-III mice per group, ***p < 0.001, **p < 0.01, *p < 0.05 vs. SOD Mock mice). Data are shown as mean ± SEM.

The results of motor nerve conduction tests showed a significant preservation of the amplitude of PL, GM and TA CMAPs in treated SOD1^G93A^ compared to mock SOD1^G93A^ control mice (Figures 2A–C). This was further corroborated by the increase in number and amplitude of remaining motor units, tested in both PL and TA muscles at the last evaluation at 16 weeks of age (Figures 2D–F). Moreover, the plot of motor units grouped by their action potential amplitude showed a higher number of the treated group in most of the intervals, reflecting a general effect on all types of motor units (Figures 2D–F). The MEPs recorded on GM and TA muscles hand also a significantly higher amplitude in the treated than in the control SOD1^G93A^ group, indicating preserved connections with the upper MNs (Figure 2G).

### Combined Gene Therapy Preserves the Number of MN and Reduces Glial Reactivity

**Figure 3:**
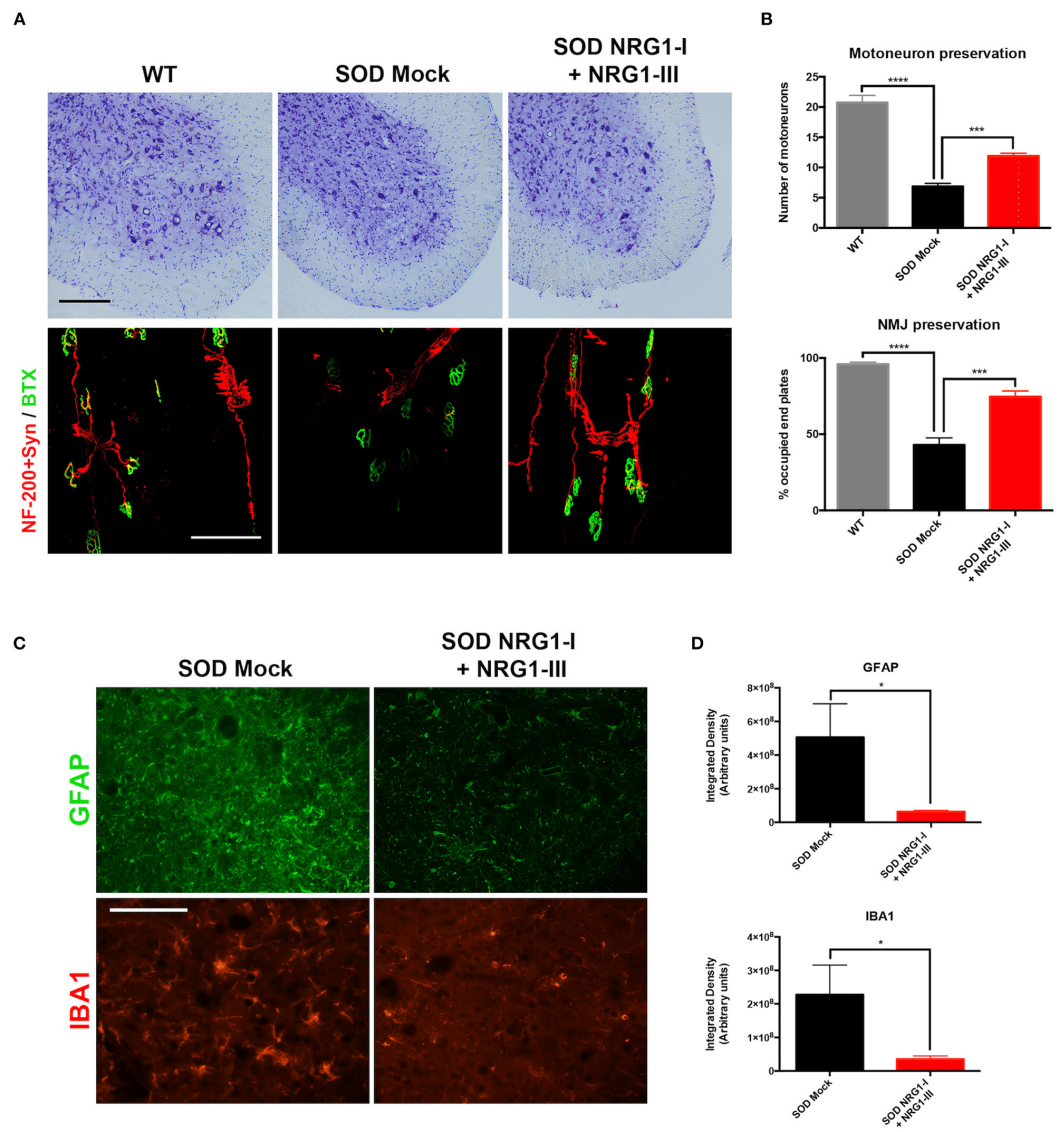
Overexpression of NRG1-I and NRG1-III preserves spinal motoneurons and reduces glial reactivity. **(A)** Representative images of L4 spinal cord and GM neuromuscular junctions of wild type and SOD1^G93A^ mice, treated with NRG1-I and NRG1-III or with mock vector (scale bar = 100 μm). **(B)** Histological analysis indicated a higher preservation of the number of spinal MNs, as well as an increased proportion of innervated NMJs in the GM muscle of the treated SOD1^G93A^ mice (*n* = 3 WT, 5 SOD Mock, 6 SOD NRG1-I + NRG1-III mice per group, ****p < 0.0001, ***p < 0.001, *p < 0.05 vs. SOD Mock mice). **(C)** Representative confocal images of astrocytes labeled with GFAP, and microglia labeled with Iba-1, in the spinal cord ventral horn of SOD1^G93A^ mice (scale bar = 100 μm). **(D)** The viral-mediated delivery of both NRG1-I and NRG1-III decreased astrocyte and microglia reactivity in the spinal cord of the SOD1^G93A^ mice (*n* = 3-4 SOD Mock, 5-6 SOD NRG1-I + NRG1-III mice per group, *p < 0.05). Data are shown as mean ± SEM.

The histological analysis performed at 16 weeks of age confirmed the improvement in neuromuscular function. There was higher number of surviving spinal MNs in the SOD1^G93A^ treated mice (11.9 ± 0.4) compared to the control SOD1^G93A^ mice (6.9 ± 0.5), and higher number of innervated NMJs (74.7 ± 3.7) than in SOD1^G93A^ mock mice (43.0 ± 4.7) (Figures 3A,B). The combined gene therapy also produced a significant decrease of astrocyte and microglial activation (6.38 × 10^7^ ± 7.18 × 10^6^ and 3.54 × 10^7^ ± 9.42 × 10^6^, respectively) whereas the untreated mice showed marked glial reactivity in the ventral horn (5.05 × 10^8^ ± 1.99 × 10^8^ and 2.27 × 10^8^ ± 8.86 × 10^7^, respectively) (Figures 3C,D).

### NRG1 Type I and Type III Isoforms Signaling Pathways

**Figure 4:**
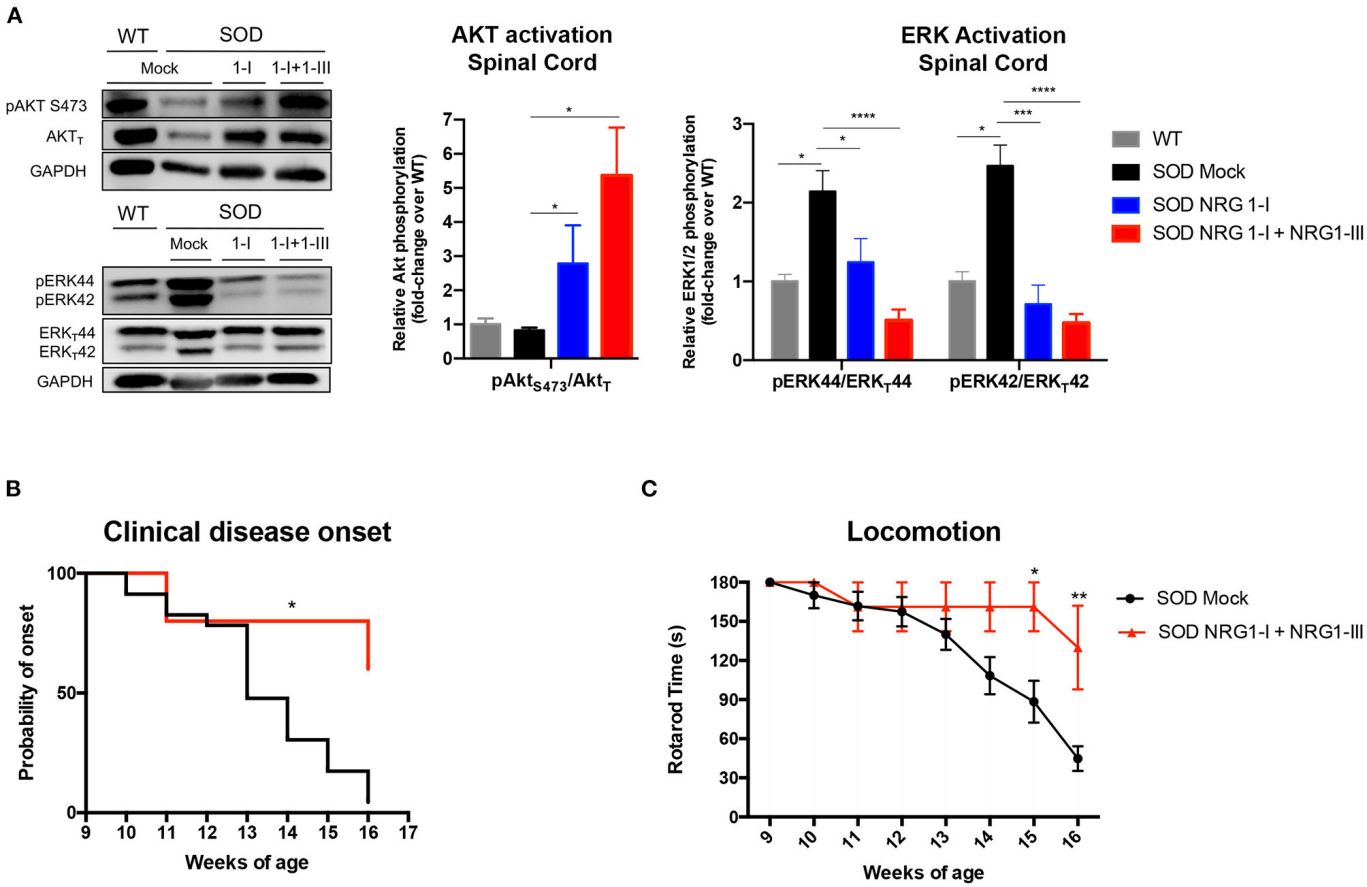
Combined gene therapy overexpressing NRG1-I and III activates survival pathways in spinal cord and produces functional improvement. **(A)** NRG1-I and combined with type III overexpression increases AKT phosphorylation and diminishes Erk2 activation in the spinal cord of the treated mice, as demonstrated by western blot. Quantifications show relative phosphorylation compared to total protein, normalized by GAPDH and are represented by fold-change compared to WT animals; (*n* = 3–5 WT mice, *n* = 6-9 SOD Mock, *n* = 4-7 SOD NRG1-I mice, *n* = 4-6 SOD NRG1-I + NRG1-III mice per group, ****p < 0.0001, ***p < 0.001, **p < 0.01 vs. SOD Mock mice). **(B)** The clinical onset of disease was significantly delayed by the combined therapy (*n* = 14 SOD Mock, 5 SOD NRG1-I + NRG1-III mice per group, Mantel-Cox test, **p < 0.01, *p < 0.05 vs. SOD Mock mice). **(C)** NRG1-I and III overexpression produced an improvement in the Rotarod performance of the treated SOD1^G93A^ mice from 14 to 16 weeks (*n* = 14 SOD Mock, 5 SOD NRG1-I + NRG1-III mice per group, **p < 0.01, *p < 0.05 vs. SOD Mock mice). Data are shown as mean ± SEM.

We then evaluated whether the overexpression of NRG1 isoforms activated downstream cell survival signaling pathways. We found that treatment with the combined gene therapy strongly upregulated Akt phosphorylation in the spinal cord compared to SOD1^G93A^ untreated mice (Figure 4A). In contrast, activation of Erk was significantly decreased in the SOD1^G93A^ treated mice, in accordance with our previous reports (40, 43).

In correspondence with the above results, we found that NRG1-I and NRG1-III overexpression produced a global functional improvement as shown in the rotarod test and delayed the clinical disease onset (Figures 4B,C).

### Combined NRG1 Gene Therapy Does Not Produce a Synergic Effect on Neuromuscular Function in SOD1^G93A^ Mice

**Figure 5:**
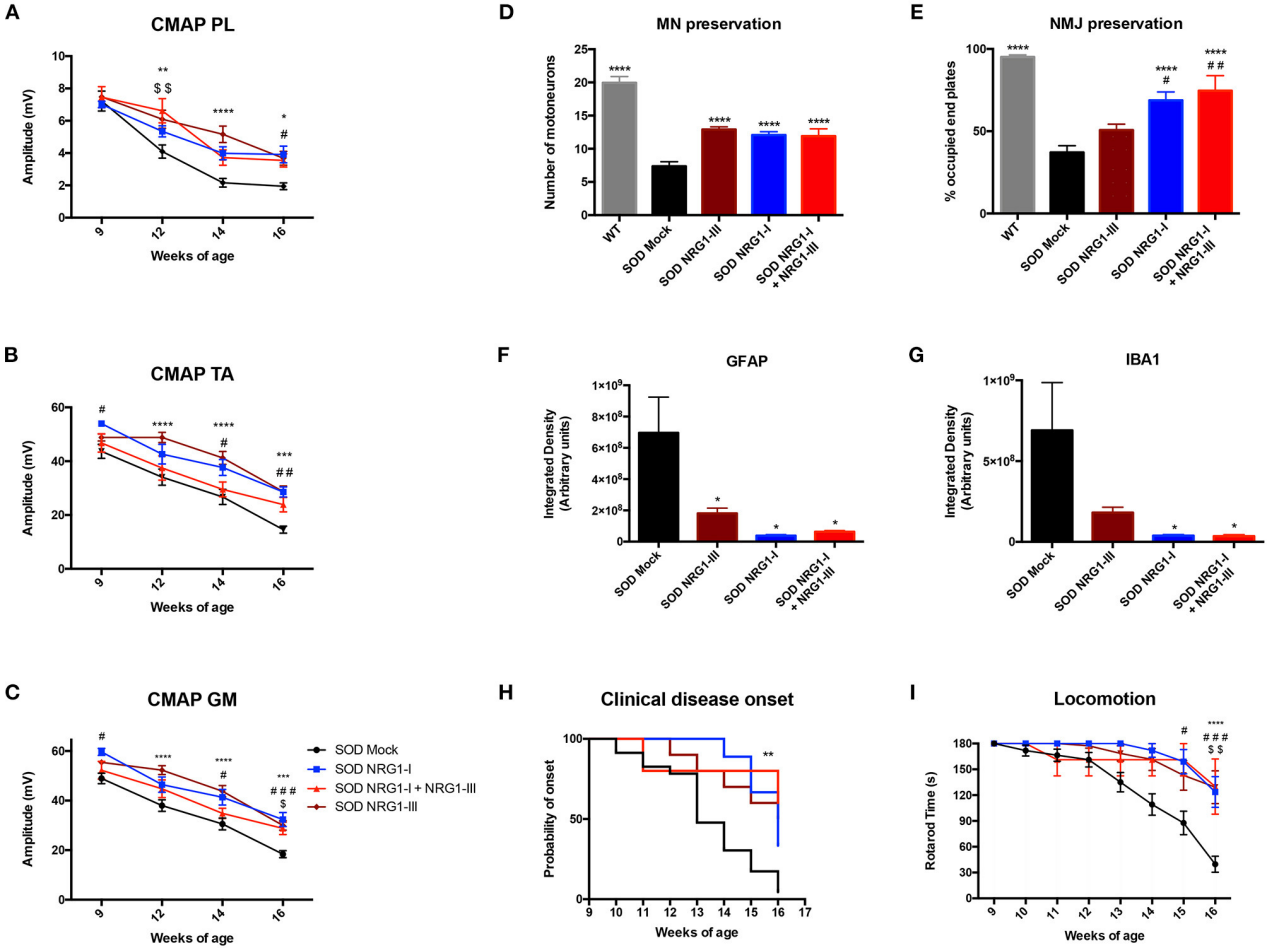
Combined gene therapy overexpressing NRG1-I and III does not promote a synergic effect. **(A)** Electrophysiological tests show that the combined gene therapy overexpressing NRG1 isoforms I and III does not improve the preservation of the CMAP amplitude of PL **(A)**, TA **(B)** and GM **(C)** compared to both gene therapies performed alone in SOD1^G93A^ mice (18-31 SOD Mock, 7-9 SOD NRG1-I + NRG1-III, 10-11 SOD NRG1-I and 20 SOD NRG1-III mice per group, ****p < 0.0001, ***p < 0.001, *p < 0.05 vs. SOD NRG1-III mice; ^###^*p* < 0.001, ^#^*p* < 0.05 vs. SOD NRG1-I mice; ^$$^*p* < 0.01, ^$^*p* < 0.05 vs. SOD NRG1-I + NRG1-III mice). **(D)** Histological analysis shows similar numbers of spinal MNs in the different treated SOD1^G93A^ mice, **(E)** but a significant increase on the proportion of innervated NMJs in the GM muscle for the combined NRG1 therapy and the NRG1-I single therapy compared to the NRG1-III alone (*n* = 6-7 WT, 8 SOD Mock, 6 SOD NRG1-I + NRG1-III, 7 SOD NRG1-I and 5-7 SOD NRG1-III mice per group, ****p < 0.0001 *p < 0.05 vs. SOD Mock mice; ^##^*p* < 0.01, ^#^*p* < 0.05 vs. SOD NRG1-III mice). **(F)** The viral-mediated delivery of the different gene therapies combining NRG1 isoforms decreased astrocyte **(G)** and microglia reactivity in the spinal cord of the SOD1^G93A^ mice (*n* = 6–8 SOD Mock, 8 SOD Mock, 5-6 SOD NRG1-I + NRG1-III, 6 SOD NRG1-I and 8 SOD NRG1-III mice per group, T-student, *p < 0.05 vs. SOD Mock mice). **(H)** The clinical onset of disease was significantly delayed by the gene therapies targeting NRG1 (*n* = 23 SOD Mock, 5 SOD NRG1-I + NRG1-III, 9 SOD NRG1-I and 10 SOD NRG1-III mice per group; Mantel-Cox test, **p < 0.01, *p < 0.05 vs. SOD Mock mice). **(I)** NRG1-I and III overexpression produced a similar improvement in the Rotarod performance of the treated SOD1^G93A^ mice compared to the two gene therapies alone (*n* = 23 SOD Mock, 5 SOD NRG1-I + NRG1-III, 7 SOD NRG1-I and 10 SOD NRG1-III mice per group, ****p < 0.0001, *p < 0.05 vs. NRG1-III mice; ^#^*p* < 0.05 vs. SOD NRG1-I mice; ^*$$*^*p* < 0.01, ^$^*p* < 0.05 vs. SOD NRG1-I + NRG1-III mice). Data are shown as mean ± SEM.

Finally, we compared the functional and histological results obtained by the combined gene therapy with those previously reported with NRG1-III (40) or NRG1-I (43) single gene therapy. The electrophysiological results showed that the combined therapy did not increase the CMAP amplitude of PL, TA, and GM muscles when compared to the gene therapies overexpressing only NRG1-III or NRG1-I (Figures 5A–C). Consistent with these results, a similar delayed onset and improvement in the rotarod test performance was found with the three therapies (Figures 5H,I). Furthermore, the histological analysis showed that the number of spinal MNs, and the astrocyte and microglia activation were similar comparing the different SOD1^G93A^ treated mice groups (Figures 5D,F,G). Interestingly, we found that gene therapy overexpressing NRG1-I in muscles alone and combined with NRG1-III significantly increased the number of occupied NMJ whereas the NRG1-III therapy alone did not (Figure 5E), which reinforces the theory of the dying-back mechanism in ALS and suggests the need to a muscle-directed therapy, as we previously suggested with other neurotrophic factors (48).

## Discussion

The results of the present study indicate that generalized increased expression of NRG1-I in body muscles as well as overexpression of NRG1-III in the spinal cord promote neuromuscular functional improvement and increase MN survival while reducing glial cell reactivity around spinal MNs in the SOD1G93A murine model of ALS. However, these positive effects did not show a synergic effect in the transgenic SOD1^G93A^ mice compared to treatment with gene therapy overexpressing either NRG1-I or NRG1-III alone.

NRG1 isoforms are essential trophic factors due to their several functions in the development and maintenance of the nervous system (49). Importantly, NRG1/ErbB system alterations have been reported in ALS human patients (38, 50). It has been described in ALS patients as well as in the SOD1^G93A^ mice, that the expression of NRG1-III is reduced in the spinal cord in correlation with MN loss (39, 51, 52). In this sense, restoration of C-boutons number and slight improvement of survival of the SOD1^G93A^ mice was shown after viral vector inducing NRG1-III administration in the CNS (51). In addition, neuroprotective effects on MNs have been previously found by our group in organotypic cultures of the spinal cord subject to excitotoxicity (52), and that overexpression of NRG1-III in the spinal cord or of NRG1-I in the skeletal muscle of the SOD1^G93A^ mice both improve motor function and maintains the number of surviving spinal MNs (40, 43). In light of these latter results, we were encouraged to combine the two gene therapies for overexpressing NRG1-I and NRG1-III to produce a synergic effect on the SOD1^G93A^ mice. Our hypothesis was that increasing production of NRG1-III at the spinal cord (by means of intrathecal administration of AAVrh10-CMV-NRG1-III) would activate protective pathways in the MNs, whereas overexpression of NRG1-I at the skeletal muscles (by means of systemic injection of AAV8-desmin-NRG1-I) would maintain the NMJ and promote re-innervation of the already denervated muscle (42). Thus, the combined gene therapy was aimed to target the two main sites of the ALS pathogenesis, the MN and the NMJ. Although published data show that dual AAV therapies injected in murine models can be successful (53, 54), this issue still presents some concerns. Administration of high-titer AAV vectors induce the generation of antibodies that could neutralize the effect of a second treatment; to avoid this, we performed both vector injections on the same day. Indeed, the injection of both viral vectors was effective, producing increased mRNA expression of NRG1-III in the spinal cord and of NRG1-I in the muscles. We previously demonstrated that intravenous injection of an AAV8 vector with the desmin promoter is able to transduce most skeletal muscles and the heart in SOD1^G93A^ mice (48). Furthermore, the results showed that the combined-gene therapy promoted functional improvement of the SOD1^G93A^ mice, although it did not achieve the expected synergic effect. One of the possible explanations could be regarding the limitations of the used model. Indeed, very few studies have reported synergistic effects after combinatory therapies (55). This might reflect that there is an endogenous limitation for the beneficial outcomes that can be achieved using the SOD1^G93A^ mouse model. In view of the past and recent results, NRG1-I and NRG1-III may differ in their functions in the nervous system but share positive effects in terms of MN survival and inflammation when applied in the peripheral and central nervous system, respectively. However, the beneficial actions of these isoforms might overlap somehow in the pathways activated in the MNs, since the combined gene therapy did not show any further positive result compared with both gene therapies alone in the mice. Certainly, NRG1-I activates the PI3K/AKT and ERK pathways when overexpressed in the GM muscle of the SOD1^G93A^ mice (42), whereas NRG1 treatment also activates the same pathways in the spinal cord to promote MN survival (52, 56). Consequently, the maximum effect that can be reached by the overexpression of NRG1 isoforms may be limited. We cannot discard, though, that a synergistic effect in spinal cord and muscle is already exerted by NRG1-III through drainage to the periphery of AAVrh10-NRG1-III vector as part of the CSF circulation, and since we used a ubiquitous promoter for this approach. Indeed, we detected slight increase of NRG1-III expression in GM muscle, although 4–5 times lower than when using the muscle specific promoter.

Regarding sex differences, it is worth to note that in our previous studies we showed that gene therapy targeting NRG1-I produced positive effects in both male and female mice, but in contrast, gene therapy for NRG1-III in the spinal cord only produced beneficial effects in the female SOD1^G93A^ mice (40, 43). Therefore, for the combined NRG1 gene therapy we used female mice, corroborating the improvements induced by NRG1. Progesterone may participate in modulating NRG1-III expression in the spinal cord and play a role on the neuroprotective effects observed in female mice (57, 58). On the other hand, this mouse model shows more severe symptoms and earlier manifestations in males than in females (59). Consequently, therapeutic treatments are often more successful in females than in males (60).

In summary, the combined NRG1-I and III overexpression significantly improved motor function preservation and promoted neuro-protection, accompanied by reduction of neuro-inflammatory reaction. However, we did not see an improved effect compared to the NRG1-I or III overexpression alone. Further studies evaluating the long-term effect of NRG1 gene therapy may help to determine its consideration for the translation to treat ALS patients.

## Data Availability Statement

The original contributions presented in the study are included in the article/Supplementary Material, further inquiries can be directed to the corresponding authors.

## Ethics Statement

The animal study was reviewed and approved by Ethics Committee of the Universitat Autònoma de Barcelona.

## Author Contributions

XN and AB conceived the funded projects and the studies. GM-C, MH-G, and NH performed the *in vivo* experiments and histological analyses. BG-L, IF-Q, and RL-V contributed to interventions and injections of viral vectors. SV and BG-L performed molecular analyses. GM-C, MH-G, AB, and XN contributed to the analyses and discussion of the results. GM-C and MH-G drafted the first version of the paper. All the authors contributed to writing and correcting the manuscript.

## Funding

This work was funded by grant TV3201428-10 of Fundació La Marato-TV3, grant #20289 of AFM-Telethon, cooperative project 2015-01 from CIBERNED, PID2020-116735RB-I00 from MICINN and TERCEL (RD16/0011/0014) funds from the Instituto de Salud Carlos III of Spain.

## Conflict of Interest

The authors declare that the research was conducted in the absence of any commercial or financial relationships that could be construed as a potential conflict of interest.

## Publisher's Note

All claims expressed in this article are solely those of the authors and do not necessarily represent those of their affiliated organizations, or those of the publisher, the editors and the reviewers. Any product that may be evaluated in this article, or claim that may be made by its manufacturer, is not guaranteed or endorsed by the publisher.

## Supplementary Material

Supplementary Figure 1 Time-chart of the gene therapy administration and the follow-up of the SOD1^G93A^ mice along the course of the disease. Elements of the figure were modified from SMART (Servier Medical Art), licensed under a Creative Common Attribution 3.0 Generic License http://smart.servier.com/.

Supplementary Figure 2 **(A)** NRG1-III mRNA expression analysis in the GM muscle. NRG1-III mRNA is also increased, by 6-fold the WT levels, probably due to virus drainage to circulation from the CSF. However, note that NRG1-I expression driven from the desmin promoter induces a 40-times increase of NRG1-I in the GM muscle (see Figure 1A). **(B)** On the contrary, no statistically significant changes in NRG1-I isoform are detected in the spinal cord between treated or non-treated SOD1^G93A^ animals, as expected, since neither AAV8 crosses the BBB nor the desmin promoter expresses in spinal cord tissue. *N* = 3 WT, 3-4 SOD Mock, 4-5 SOD NRG1-I + NRG1-III mice per group. One-way ANOVA, ^*^*p* < 0.05 vs. SOD Mock group.
