# Supplementary figures and images for: Gene Therapy Overexpressing Neuregulin 1 Type I in Combination With Neuregulin 1 Type III Promotes Functional Improvement in the SOD1G93A ALS Mice
Source: Front Neurol. 2021 Sep 22;12:693309. doi: 10.3389/fneur.2021.693309 (PMC8492910; doi:10.3389/fneur.2021.693309)

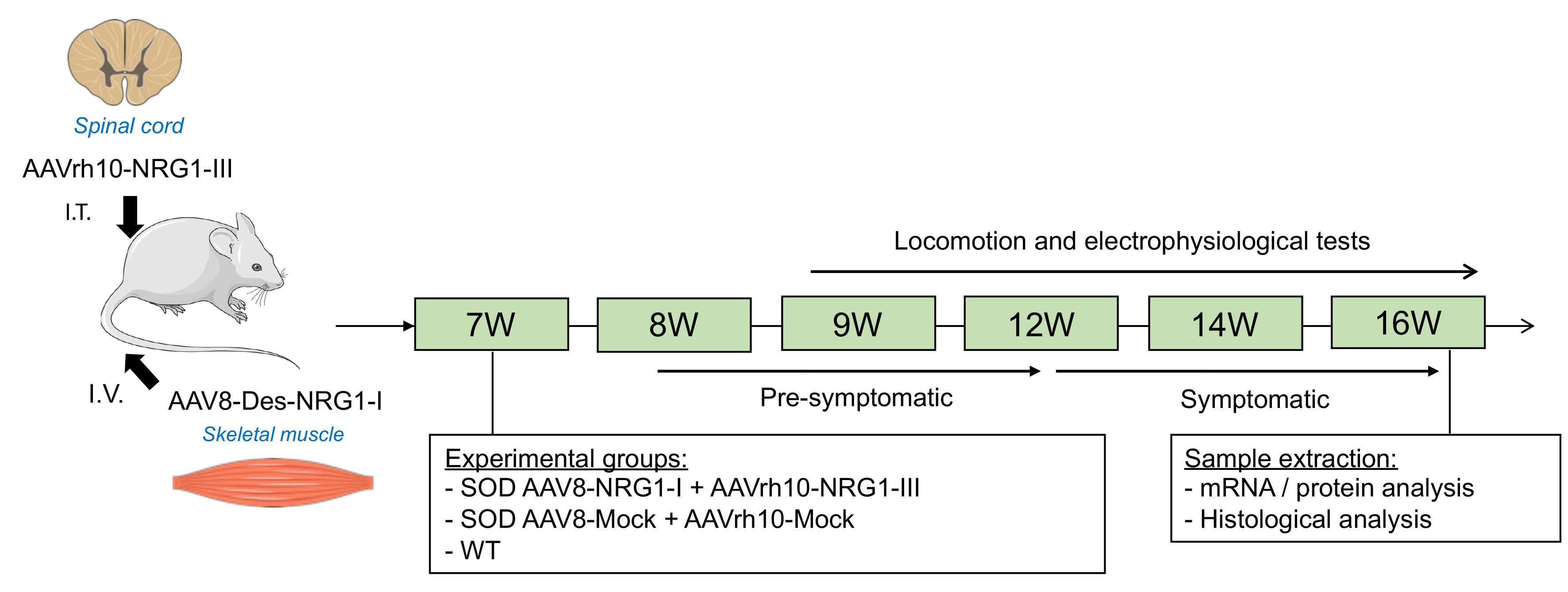

Supplement: Supplementary Figure 1 — Time-chart of the gene therapy administration and the follow-up of the SOD1G93A mice along the course of the disease. Elements of the figure were modified from SMART (Servier Medical Art), licensed under a Creative Common Attribution 3.0 Generic License http://smart.servier.com/. [file Image_1.TIF]

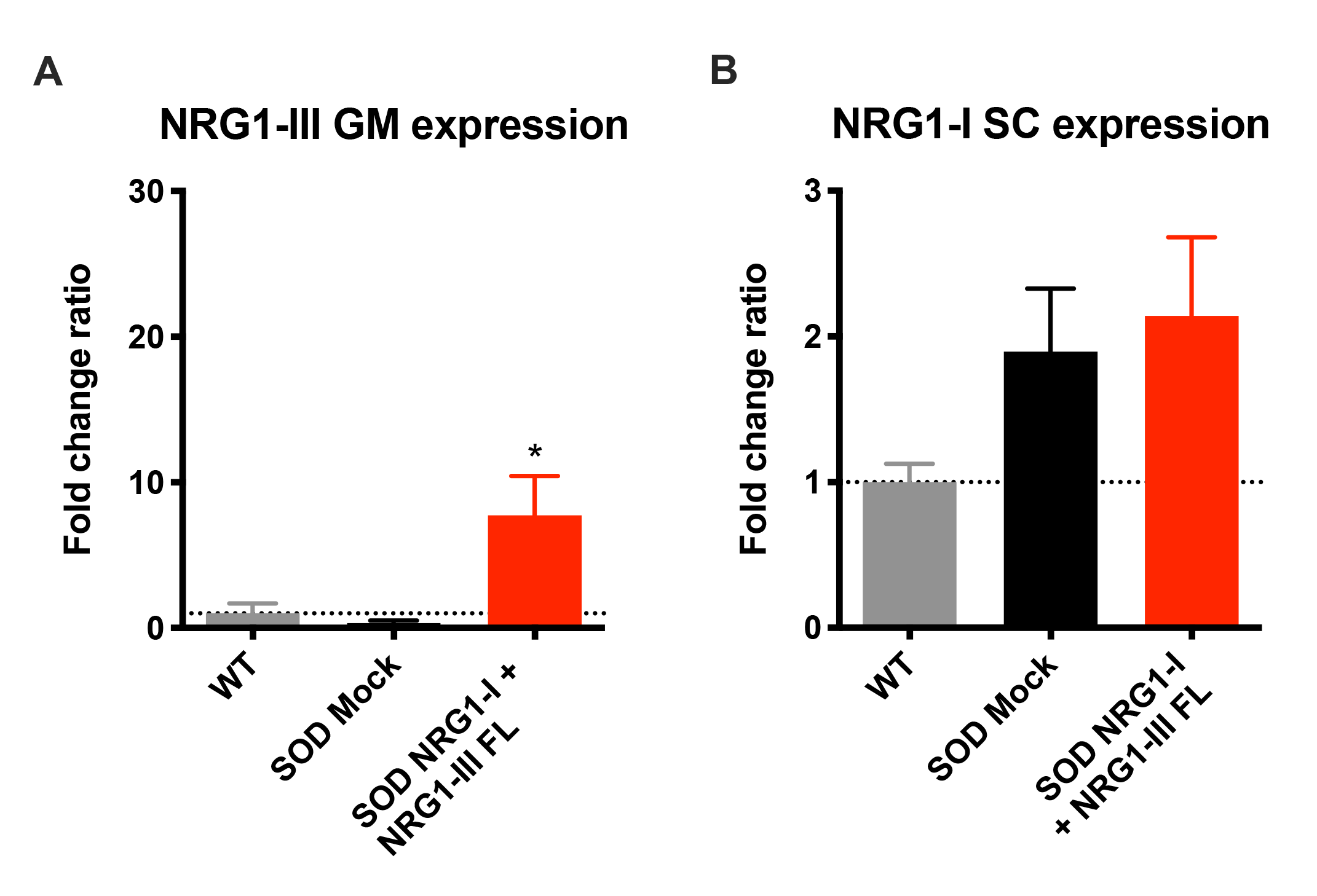

Supplement: Supplementary Figure 2 — (A) NRG1-III mRNA expression analysis in the GM muscle. NRG1-III mRNA is also increased, by 6-fold the WT levels, probably due to virus drainage to circulation from the CSF. However, note that NRG1-I expression driven from the desmin promoter induces a 40-times increase of NRG1-I in the GM muscle (see Figure 1A). (B) On the contrary, no statistically significant changes in NRG1-I isoform are detected in the spinal cord between treated or non-treated SOD1G93A animals, as expected, since neither AAV8 crosses the BBB nor the desmin promoter expresses in spinal cord tissue. N = 3 WT, 3-4 SOD Mock, 4-5 SOD NRG1-I + NRG1-III mice per group. One-way ANOVA, *p < 0.05 vs. SOD Mock group. [file Image_2.TIF]
